# Coagulation parameter-based nomogram for the diagnosis of obstetric antiphospholipid syndrome and its subtypes

**DOI:** 10.1515/biol-2025-1252

**Published:** 2026-02-12

**Authors:** Xuan Qi, Feifei Zhang, Xiaomeng Li, Yan Han, Yue Zhang, Huifang Guo

**Affiliations:** Department of Rheumatology and Immunology, The Second Hospital of Hebei Medical University, Shijiazhuang 050000, Hebei, China; Special Examination Department, The Second Hospital of Hebei Medical University, Shijiazhuang 050000, Hebei, China; Department of Fertility, The Second Hospital of Hebei Medical University, Shijiazhuang 050000, Hebei, China

**Keywords:** obstetric antiphospholipid syndrome, diagnostic model, risk factors, thrombin-antithrombin complex, coagulation factor activity

## Abstract

Currently available classification criteria for obstetric antiphospholipid syndrome (OAPS) are often overly strict and may miss a considerable number of patients with so-called “non-criteria-defined OAPS”. We aimed to establish a diagnostic nomogram based on clinical and laboratory parameters to facilitate the diagnostic efficacy for OAPS by incorporating both criteria – and non-criteria – defined OAPS. We retrospectively analyzed the clinical and laboratory data of 45 patients with criteria-defined OAPS, 57 with non-criteria-defined OAPS, and 80 age-matched healthy controls between September 2023 and March 2024. We established a nomogram for OAPS based on the risk factors identified through logistic regression and evaluated its performance using the receiver-operating characteristic curve, calibration curve, and decision curve analysis. Antinuclear antibody titer, as well as levels of complement C3, anti-β2 glycoprotein I, thrombin-antithrombin complex, and Von Willebrand factor were independent risk factors for OAPS (*P* < 0.05). In the training set, the nomogram established using these variables exhibited an area under the curve of 0.97, a sensitivity of 89 %, and a specificity of 94 %. In the validation set, these values were 0.99, 100 % and 88 %, respectively. The nomogram demonstrated enhanced diagnostic capabilities and facilitated more precise treatment guidance for OAPS.

## 1 Introduction

Antiphospholipid syndrome (APS) is a systemic autoimmune disease orchestrated by antiphospholipid antibodies, with clinical manifestations of vascular thrombosis and recurrent obstetric events [1], 2]. The obstetric APS (OAPS) represents a subset of APS that poses a significant threat to maternal and infant health by incurring early pregnancy loss, fetal demise in the second and third trimesters, and preterm delivery related to preeclampsia or placental insufficiency [3]. Thus, early and accurate diagnosis is critical for the improvement of obstetric outcomes in OAPS.

The current classification criteria for OAPS are still based on the 1999 Sapporo classification criteria [4] and the 2006 Sydney Revised APS classification criteria [5]. In the latest 2023 APS classification proposed by the European League Against Rheumatism/American College of Rheumatology [6], a diagnosis of APS requires one laboratory criterion within 3 years of the identification of a clinical criterion, plus at least three points from each of the six clinical and two laboratory domains. The diagnostic and classification criteria are often unachievable in clinical practice and thus may be more applicable to scientific research [7]. For instance, a definitive diagnosis of OAPS typically requires the presence of antiphospholipid antibodies or a specific thromboembolic event, which may prove to be overly restrictive for clinical diagnosis. Specifically, the Sydney clinical criteria stipulate at least three consecutive miscarriages before 10 weeks of gestation, at least one fetal loss before 10 weeks of gestation, at least one preterm delivery before 34 weeks of gestation due to preeclampsia or eclampsia or placental insufficiency [5]. Laboratory criteria for OAPS includes two positive tests for lupus anticoagulant 12 weeks apart, two immunoglobulin M (IgM) or G (IgG) tests for anticardiolipin (ACL) antibodies 12 weeks apart, two IgM/IgG tests for anti-β2-glycoprotein I (aβ2-GPI) antibodies 12 weeks apart [5]. Although this new criteria has been demonstrated to have a sensitivity of 73 % and specificity of 94 % for the diagnosis of APS [8], it is not specifically designed for OAPS, and many patients with only typical clinical manifestations or only high anti-phospholipid antibodies would be misclassified [9]. Moreover, the heterogeneous clinical manifestations of OAPS may result in some atypical symptoms being overlooked by existing classification criteria, potentially leading to delayed diagnosis and treatment in some patients [10]. In view of these limitations, the potential importance of non-classical clinical or laboratory manifestations of OAPS is being increasingly recognized with a better understanding of the pathogenesis and heterogeneous clinical features [11]. For instance, approximately 27.34 % of non-criteria-defined OAPS did not meet laboratory criteria and 45.15 % of patients did not meet clinical criteria as required by the 2006 Sydney criteria [12]. Thus, the inclusion of non-criteria-defined OAPS in the development of a diagnostic model for OAPS is crucial for the early identification and prediction of high-risk populations, improvement of pregnancy outcomes and reduction of adverse pregnancy events.

A clinicopathologic hallmark of APS is thrombo-inflammation that occurs at the endothelial surface and is driven by circulating autoantibodies that recognize cell surface phospholipids and phospholipid-binding proteins [13]. Pregnancy is a hypercoagulable state, in which the procoagulant activity increases, while the anticoagulant activity decreases [14]. Therefore, hyper coagulability and an elevated level of coagulated factors are found in women with OAPS, which further leads to life-threatening complications or adverse pregnancy outcomes [15], 16].

To improve diagnostic accuracy and clinical utility, new diagnostic tools and models should be developed or revised to optimize the current OAPS classification criteria. Therefore, the present study aimed to construct and validate a diagnostic nomogram for OAPS by including both criteria- and non-criteria-defined OAPS. This diagnostic tool might be a potential predictive tool for the occurrence of OAPS during pregnancy, and thereby performing rapid treatment to improve pregnancy outcomes.

## 2 Methods

### 2.1 Study design

This study is a single-center retrospective cohort analysis at the Department of Rheumatology and Immunology, the Second Hospital of Hebei Medical University between September 2023 and March 2024.

We retrospectively reviewed the patients’ data and collected those diagnosed with criteria-defined OAPS (criteria-defined OAPS group, *n* = 45) and non-criteria-defined OAPS (non-criteria-defined OAPS group, *n* = 57). The inclusion criteria were 1) women diagnosed with criteria- or non-criteria-defined OAPS; 2) women presented with positive antiphospholipid antibodies and recurrent abortions; and 3) women aged between 20 and 40 years old. Criteria-defined OAPS was diagnosed based on the fulfillment of at least one clinical criterion and one laboratory test criterion for pathological pregnancy, according to the 2006 revised Sydney classification criteria for OAPS [5]. Non-criteria-defined OAPS was defined based on the 2020 International Consensus Proposal for Non-Criteria OAPS proposed by Alijotas-Reig et al. [17], which encompasses patients with typical OAPS-related clinical manifestations or laboratory findings that do not fully meet the 2006 Sydney Criteria. These patients are clinically relevant because they still face increased risks of adverse pregnancy outcomes but are excluded by the strict thresholds of criteria-defined OAPS. The exclusion criteria included 1) a history of smoking; 2) medical comorbidities of hypertension, diabetes mellitus, heart disease, chronic kidney disease, liver disease, malignancy, and immune system disorders prior to or during pregnancy; 3) acute and chronic hematologic disorders prior to or during pregnancy; 4) use of anticoagulants or antiplatelet medications prior to inclusion; 5) recent history of trauma and surgery; 6) endometriosis; 7) uterine malformations, and other gynecologic conditions that predispose to miscarriage; and 8) recurrent miscarriage and fetal developmental delay due to chromosomal or genetic abnormalities in either the embryo or the parents.

Additionally, we randomly selected 80 healthy women of same age range who underwent health examinations in our hospital during the same period as healthy controls.

**Informed consent:** Informed consent has been obtained from all individuals included in this study.

**Ethical approval:** The research related to human use has been complied with all the relevant national regulations, institutional policies and in accordance with the tenets of the Helsinki Declaration, and has been approved by the Research Ethics Committee of the Second Hospital of Hebei Medical University (No. 2024-R124).

### 2.2 Data collection

We collected the following data from the patients and/or healthy controls: 1) age and reproductive history; 2) laboratory test results (Table 1). The blood tests were performed within 4–12 weeks in the first trimester.

**Table 1:** The methods for laboratory test.

Variables	Testing methods	Manufacturers	Catalog number
White blood cell count	Flow cytometry method	SYSMEX Corporation	XS-500i
Platelet count	Flow cytometry method	SYSMEX Corporation	XS-500i
Antinuclear antibody tier	Chemiluminescence method	Shenzhen Yahuilong Biotechnology Co. Ltd	iFlash3000-H
Complement C3 and C4	Immunoturbidimetric assay	Beckman Coulter, Inc.	AU680
IgG	Immunological methods	Beckman Coulter, Inc.	AU680
Anticardiolipin	Chemiluminescence method	Shenzhen Yahuilong Biotechnology Co. Ltd	iFlash3000-H
aβ2-GPI antibody	Chemiluminescence method	Shenzhen Yahuilong Biotechnology Co. Ltd	iFlash3000-H
25-Hydroxyvitamin D level	Chemiluminescence method	Shenzhen Yahuilong Biotechnology Co. Ltd	iFlash3000-H
Russell viper venom time	Coagulation method	Werfen North America	ACL TOP 750
Silica clotting time	Coagulation method	Werfen North America	ACL TOP 750
Activated partial thromboplastin time	Coagulation method	SYSMEX Corporation	CS 2400
Platelet aggregation rate	Light transmission aggregometry	SYSMEX Corporation	CS 2400
Thrombin-antithrombin complex	Chemiluminescence analysis	Wondfo Biotech	ACL TOP 750
D-dimer	Immune turbidimetry	SYSMEX Corporation	CS 2400
Coagulation factors (V, VII, VIII, X)	Instrumental method	Werfen North America	ACL TOP 750
Von Willebrand factor	Immune turbidimetry	Werfen North America	ACL TOP 750
Protein S	Coagulation method	Werfen North America	ACL TOP 750
Protein C	Chromogenic substrate method	Werfen North America	ACL TOP 750
Thromboelastography	Viscosimetry	Beijing Pro-med Technology Co., Ltd	CFMS LEPU-8880

### 2.3 Statistical analysis

We processed the data with R 4.3.1 (RStudio, Boston, MA, USA) and SPSS 21.0 (IBM, NY, USA). Categorical variables were expressed as counts (percentages), and differences between groups were compared using the chi-square test, chi-square correction test or Fisher’s exact test where applicable. Distribution of continuous variables were first checked by Shapiro–Wilk test. The data followed normal distribution were expressed as mean ± standard deviation and compared using independent Student’s *t*-test to identify significant different factors among groups. Non-normally distributed data were expressed as median (interquartile range), and differences between groups were compared using nonparametric Mann–Whitney *U* test to identify significant different factors among groups. The cohort was randomly divided into the training set (70 %) to identify risk factors for OAPS and the validation set (30 %) to validate these risk factors. The random allocation of the cohort into a training set (70 %) and a validation set (30 %) was performed using the createDataPartition function from the caret package in R (version 4.4.2). To ensure reproducibility, a random seed was set to 123 (set.seed(123)) prior to data splitting. Crucially, we employed a stratified sampling approach, using the patient subtype (criteria-defined OAPS, non-criteria-defined OAPS, and healthy controls) as the stratification variable. This method guarantees that the proportional representation of each subgroup is maintained across both the training and validation sets, thereby preventing sampling bias and enhancing the robustness of our model evaluation. Univariate logistic regression and multivariate logistic regression analyses were performed to explore the independent risk factors influencing the occurrence of OAPS. A nomogram prediction model used for predicting the occurrence of OAPS was constructed based on the identified influencing factors, and the performance and clinical utility of the nomogram model for diagnosing OAPS was analyzed using receiver-operating characteristic (ROC) curve, calibration curve and decision curve analysis. A two-sided *P* value <0.05 denoted statistical significance.

## 3 Results

### 3.1 Comparison of the training set and validation set

A total of 45 patients with criteria-defined OAPS, 57 with non-criteria-defined OAPS, and 80 age-matched healthy controls were included and divided into the training set (70 %, 127 patients, 34 criteria-defined OAPS, 36 non-criteria-defined OAPS, and 57 healthy controls) and the validation set (30 %, 55 patients, 11 criteria-defined OAPS, 21 non-criteria-defined OAPS, and 23 healthy controls). As illustrated in Table 2, except for maximum amplitude (*P* = 0.006), other parameters were not statistically significant between the training and validation set, including age, white blood cell count, platelet count, ANA, C3, C4, IgG, ACL, aβ2-GPI, 25-hydroxyvitamin D level, the Russell viper venom time, silica clotting time ratio, APTT, platelet aggregation rate, TAT, D-dimer, coagulation factors V, VII, VIII, X, Von Willebrand factor, protein S, protein C, and thromboelastography parameters R time, and coagulation index (*P* > 0.05).

**Table 2:** Comparison of baseline patient characteristics between the training set and validation set.

Variables	Training set (*n* = 127)	Validation set (*n* = 55)	*t/Z/χ* ^2^	*P*
Age, years	31.99 ± 5.50	30.73 ± 5.38	−1.43	0.154
C3, g/L	1.02 (0.82–1.17)	1.04 (0.81–1.29)	−0.52	0.600
C4, g/L	0.23 (0.17–0.34)	0.22 (0.15–0.30)	−1.23	0.220
Immunoglobulin G, g/L	13.30 (12.40–14.50)	12.70 (11.70–14.75)	−1.37	0.170
Anti-cardiolipin antibody, GPL/mL	4.04 (2.17–8.32)	3.82 (1.96–7.08)	−0.91	0.364
Anti-β2 glycoprotein I antibody, RU/mL	19.63 (13.52–38.34)	16.95 (12.48–44.45)	−0.57	0.567
Russell viper venom time	0.99 (0.91–1.06)	0.98 (0.92–1.04)	−0.08	0.939
Silica clotting time	0.98 (0.89–1.04)	0.96 (0.89–1.04)	−0.63	0.528
Platelet count, ×10^9^/L	220.00 (193.50–276.50)	215.00 (192.50–263.50)	−0.57	0.570
White blood cell count, ×10^9^/L	6.10 (5.20–7.35)	6.30 (5.05–7.70)	−0.33	0.744
Thrombin-antithrombin complex, ng/mL	4.39 (2.96–6.21)	4.55 (2.83–6.92)	−0.07	0.943
D-dimer, mg/L	0.08 (0.06–0.12)	0.09 (0.06–0.22)	−0.80	0.425
Platelet aggregation rate, %	89.90 (81.55–92.50)	89.80 (78.10–92.35)	−0.56	0.577
*R* time, min	5.10 (4.40–6.05)	4.80 (4.15–5.35)	−1.59	0.112
Maximum amplitude, mm	62.70 (58.55–66.65)	64.80 (61.40–69.00)	−2.73	0.006
Coagulation index	1.10 (0.10–2.00)	1.10 (0.20–2.30)	−0.31	0.757
Von Willebrand factor, %	105.80 (82.55–168.80)	126.90 (93.60–180.35)	−1.48	0.138
Coagulation factor VIII, %	104.40 (82.55–125.80)	112.70 (84.00–134.65)	−0.98	0.327
Coagulation factor X, %	105.50 (85.95–122.90)	104.80 (84.55–120.10)	0.00	1.000
Coagulation factor VII, %	95.30 (82.10–113.95)	91.80 (77.75–116.70)	−0.72	0.470
Coagulation factor V, %	83.40 (74.50–99.40)	89.00 (74.90–103.80)	−0.85	0.393
Activated partial thromboplastin time, s	30.60 (28.75–32.10)	30.10 (28.95–32.35)	−0.09	0.928
Protein S, %	73.00 (66.90–84.45)	76.70 (68.40–89.30)	−1.67	0.094
Protein C, %	100.20 (88.50–110.55)	98.00 (87.05–110.55)	−0.11	0.915
25-hydroxy vitamin D, ng/mL	34.70 (20.45–50.05)	45.00 (21.91–52.85)	−1.06	0.287
ANA, 0/1:100/1:320/>1:320	77/36/10/4	31/17/6/1	0.854	0.835

The pregnancy outcomes of women with non-criteria-defined OAPS and criteria-defined OAPS are summarized in Table 3. The percentages of fetal death, preeclampsia and preterm birth were comparable between criteria-defined OAPS group and non-criteria-defined OAPS group (*P* > 0.05).

**Table 3:** Pregnancy outcomes of non-criteria-defined OAPS and criteria-defined OAPS.

Variables	Non-criteria-defined OAPS (*n* = 57)	Criteria-defined OAPS (*n* = 45)	*χ* ^2^	*P*
Fetal death	5 (8.8 %)	13 (28.9 %)	7.003	0.008
Preeclampsia*	1 (1.8 %)	1 (2.2 %)		0.866
Preterm birth	2 (3.5 %)	2 (4.4 %)	0	1.000

*P value was calculated using Fisher’s exact test.

### 3.2 Comparison between the OAPS group and healthy controls in the training set

The comparison between the two groups is depicted in Table 4. Compared with the healthy controls, the OAPS group had significantly lower levels of C3, C4, coagulation factor V, protein S, as well as a significantly higher positive rate of ANA, elevated levels of aβ2-GPI, TAT, platelet aggregation rate, coagulation index, APTT, coagulation factor VIII, and Von Willebrand factor (*P* < 0.05).

**Table 4:** Comparison between the OAPS group and healthy controls in the training set.

Variables	OAPS (*n* = 70)	Healthy control (*n* = 57)	*t/Z*	*P*
Age, years	32.19 ± 4.58	31.75 ± 6.50	−0.423	0.673
C3, g/L	0.96 (0.78–1.10)	1.12 (0.92–1.29)	−3.879	<0.001
C4, g/L	0.21 (0.16–0.28)	0.33 (0.19–0.40)	−3.140	0.002
Immunoglobulin G, g/L	13.30 (12.12–14.40)	13.40 (12.50–14.50)	−0.323	0.747
Anti-cardiolipin antibody, GPL/mL	3.28 (2.07–8.62)	4.69 (2.91–8.05)	−1.251	0.211
Anti-β2 glycoprotein I antibody, RU/mL	30.45 (20.12–56.53)	14.97 (12.30–18.41)	−5.773	<0.001
Russell viper venom time	0.99 (0.91–1.07)	0.99 (0.91–1.06)	−0.507	0.612
Silica clotting time	0.96 (0.89–1.02)	0.99 (0.89–1.10)	−0.667	0.505
Platelet count, ×10^9^/L	230.00 (204.00–281.50)	199.00 (192.00–255.00)	−2.070	0.038
White blood cell count, ×10^9^/L	6.10 (5.10–7.10)	6.30 (5.50–7.40)	−0.141	0.888
Thrombin-antithrombin complex, ng/mL	5.93 (4.72–8.84)	2.89 (2.12–3.85)	−7.896	<0.001
D-dimer, mg/L	0.08 (0.06–0.11)	0.08 (0.05–0.27)	−0.515	0.606
Platelet aggregation rate, %	92.05 (89.35–93.18)	82.40 (76.60–88.90)	−5.609	<0.001
R time, min	5.20 (4.80–5.60)	4.50 (3.90–6.70)	−1.276	0.202
Maximum amplitude, mm	63.70 (60.90–66.70)	61.40 (57.20–66.00)	−2.402	0.016
Coagulation index	1.30 (0.53–1.90)	0.90 (−0.70–2.30)	−1.319	0.187
Von Willebrand factor, %	135.10 (92.30–184.75)	90.00 (70.80–124.80)	−3.905	<0.001
Coagulation factor VIII, %	110.20 (91.88–141.75)	96.80 (70.40–116.10)	−3.008	0.003
Coagulation factor X, %	105.15 (88.12–124.67)	105.60 (84.80–120.70)	−0.810	0.418
Coagulation factor VII, %	94.20 (81.65–112.92)	102.20 (82.50–117.80)	−1.081	0.280
Coagulation factor V, %	80.85 (69.70–92.97)	88.70 (79.30–111.80)	−3.837	<0.001
Activated partial thromboplastin time, sec	31.30 (29.80–32.20)	29.30 (27.80–31.30)	−3.792	<0.001
Protein S, %	70.30 (60.23–78.78)	76.20 (67.80–87.20)	−2.880	0.004
Protein C, %	104.50 (90.67–110.00)	97.40 (85.50–110.90)	−1.142	0.254
25-Hydroxyvitamin D, ng/mL	34.75 (20.22–48.55)	34.30 (22.10–53.00)	−0.395	0.693
ANA*, 0/1:100/1:320/>1:320	26/32/8/4	51/4/2/0		<0.001

*P value was calculated by Fisher’s exact test.

### 3.3 Logistic regression analysis

As demonstrated in Table 5, univariate binary logistic regression analysis indicated that ANA, C3, C4, aβ2-GPI, TAT, platelet aggregation rate, coagulation factor VIII & V, APTT, Von Willebrand factor and protein S were factors associated with a diagnosis of OAPS (*P* < 0.05). Subsequent multivariate logistic regression analysis revealed that ANA, C3, aβ2-GPI, TAT, and Von Willebrand factor were independently associated with a diagnosis of OAPS (*P* < 0.05). These independent factors might be potential biomarkers for diagnosing OAPS.

**Table 5:** Results of univariate and multivariate logistic regression for obstetric antiphospholipid syndrome.

Variables	Univariate analysis					Multivariate analysis				
	*β*	S.E	Wald *χ* ^2^	OR (95 % CI)	*P*	*β*	S.E	Wald *χ* ^2^	OR (95 % CI)	*P*
Anti-nuclear antibody titer			26.09		<0.001					
1:100	2.75	0.58	22.34	15.69 (5.01–49.15)	<0.001	2.48	1.20	2.06	11.93 (1.13–125.60)	0.039
1:320	2.06	0.83	6.21	7.85 (1.55–39.64)	0.013	1.00	1.37	0.72	2.71 (0.18–40.03)	0.469
>1:320	21.88	20,096.49	0.00	3,168,816,080.51 (0.00–Inf)	0.999	12.54	1,646.21	0.01	278,025.77 (0.00–Inf)	0.994
Age, years	0.01	0.03	0.19	1.01 (0.95–1.08)	0.659					
C3, g/L	−3.47	0.88	15.37	0.03 (0.01–0.18)	<0.001	−3.09	1.84	1.68	0.05 (0.00–1.69)	0.044
Immunoglobulin G, g/L	0.03	0.08	0.18	1.03 (0.89–1.20)	0.673					
Anti-cardiolipin antibody, GPL/mL	0.02	0.02	1.35	1.02 (0.99–1.06)	0.246					
C4, g/L	−4.40	1.61	7.42	0.01 (0.00–0.29)	0.006	−2.65	2.63	1.01	0.07 (0.00–12.29)	0.314
Anti-β2 glycoprotein I antibody, RU/mL	0.10	0.02	18.09	1.10 (1.05–1.15)	<0.001	0.06	0.03	2.21	1.07 (1.01–1.13)	0.027
Silica clotting time	0.11	0.87	0.02	1.12 (0.20–6.16)	0.901					
Russell viper venom time	0.67	0.78	0.74	1.95 (0.43–8.97)	0.389					
White blood cell count, ×10^9^/L	0.05	0.11	0.22	1.06 (0.84–1.32)	0.638					
Thrombin-antithrombin complex, ng/mL	0.99	0.19	27.73	2.70 (1.86–3.90)	<0.001	0.89	0.26	3.36	2.43 (1.45–4.07)	<0.001
Platelet count, ×10^9^/L	0.00	0.00	1.24	1.00 (1.00–1.01)	0.266					
D-dimer, mg/L	−0.14	0.24	0.35	0.87 (0.54–1.39)	0.555					
*R* time, min	0.03	0.14	0.05	1.03 (0.79–1.35)	0.830					
Maximum amplitude, mm	0.04	0.03	1.51	1.04 (0.98–1.10)	0.219					
Platelet aggregation rate, %	0.10	0.03	11.93	1.10 (1.04–1.16)	<0.001	0.03	0.04	0.68	1.03 (0.95–1.12)	0.497
Coagulation index	0.15	0.12	1.50	1.16 (0.92–1.47)	0.221					
Coagulation factor VIII, %	0.02	0.01	8.66	1.02 (1.01–1.03)	0.003	0.00	0.02	0.24	1.00 (0.97–1.03)	0.809
Coagulation factor X, %	0.01	0.01	1.58	1.01 (0.99–1.02)	0.209					
Von Willebrand factor, %	0.01	0.00	13.11	1.01 (1.01–1.02)	<0.001	0.02	0.01	1.98	1.02 (1.01–1.04)	0.048
Coagulation factor VII, %	−0.00	0.01	0.30	1.00 (0.98–1.01)	0.587					
Activated partial thromboplastin time, sec	0.20	0.08	6.94	1.23 (1.05–1.43)	0.008	−0.04	0.14	0.27	0.96 (0.73–1.27)	0.785
Coagulation factor V, %	−0.05	0.01	15.88	0.96 (0.93–0.98)	<0.001	−0.02	0.03	0.61	0.98 (0.94–1.04)	0.539
Protein S, %	−0.02	0.01	4.95	0.98 (0.96–0.99)	0.026	−0.00	0.02	0.10	1.00 (0.96–1.04)	0.918
Protein C, %	0.01	0.01	1.85	1.01 (0.99–1.03)	0.174					
25-Hydroxyvitamin D, ng/mL	−0.00	0.01	0.09	1.00 (0.98–1.02)	0.770					

### 3.4 Nomogram construction and validation

**Figure 1:**
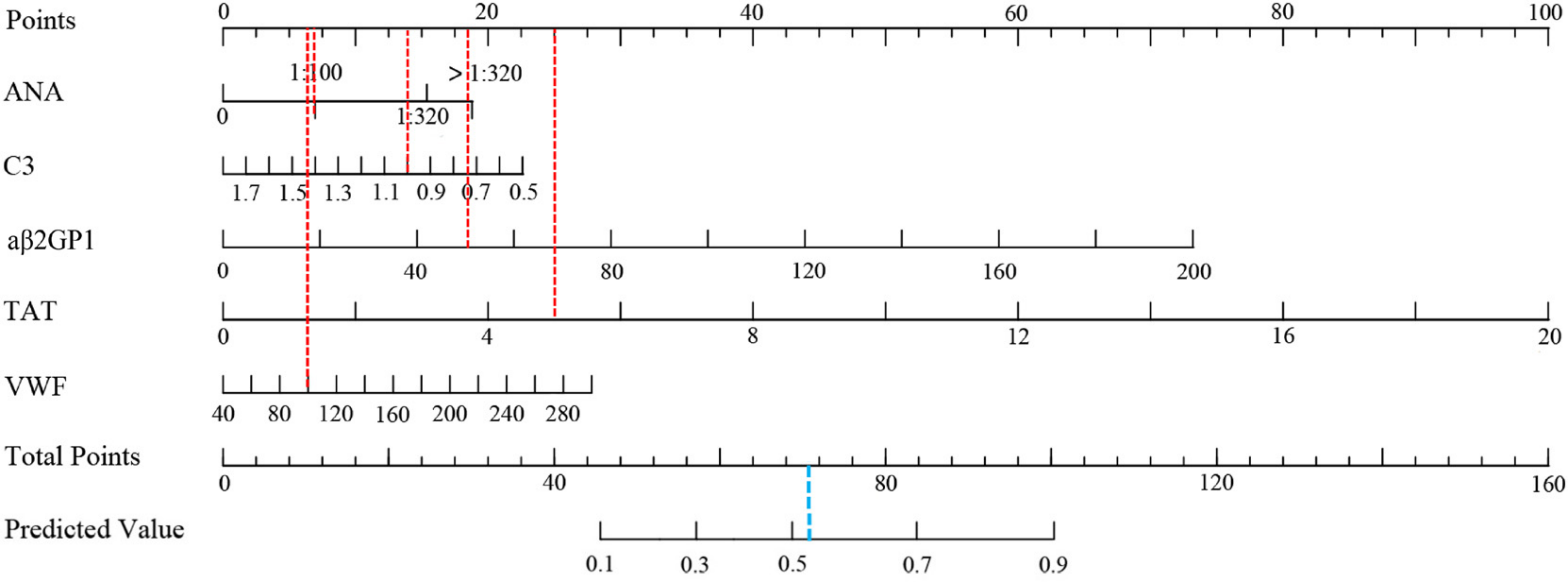
Nomogram predicting the probability of obstetric antiphospholipid syndrome. ANA, antinuclear antibody; aβ2-GPI, anti-β2 glycoprotein I antibody; TAT, thrombin-antithrombin complex; VWF, Von Willebrand factor.

**Figure 2:**
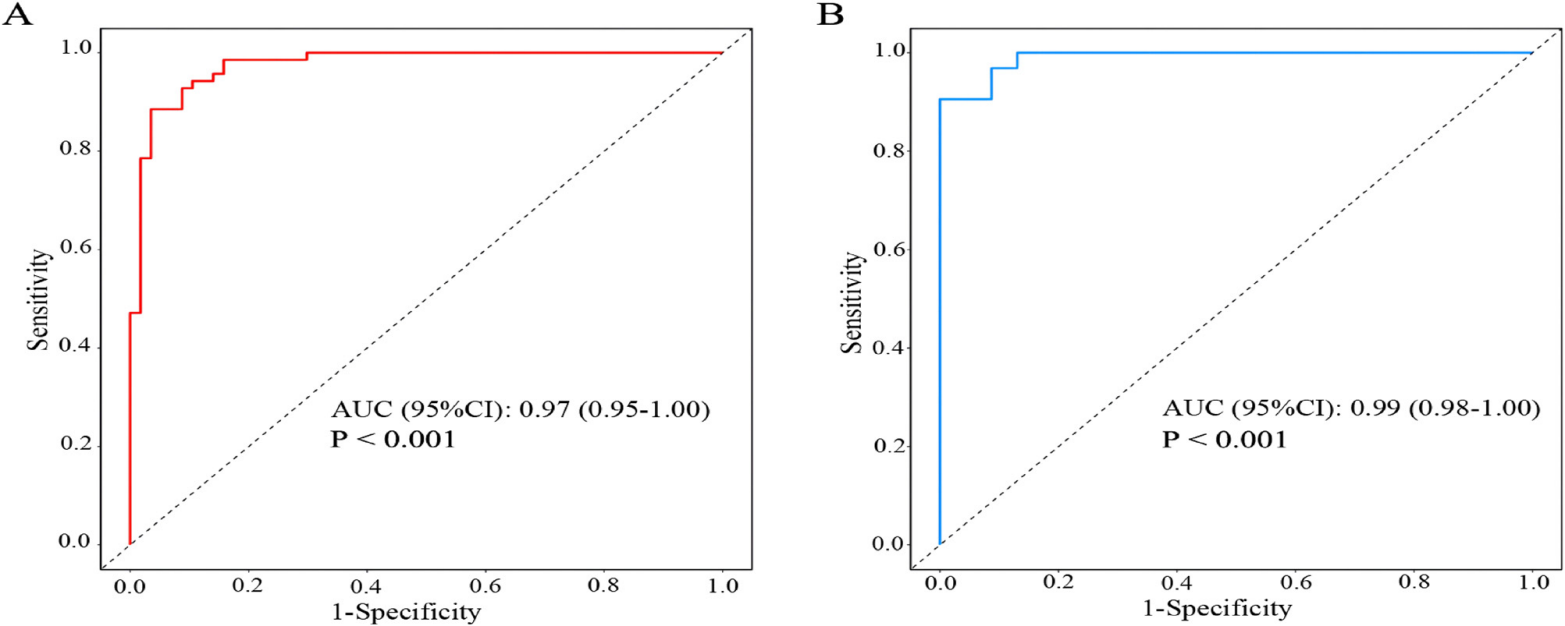
Receiver-operating characteristic curves for the discrimination of obstetric antiphospholipid syndrome from healthy controls in the training set (A) and validation set (B). AUC, area under the curve.

**Figure 3:**
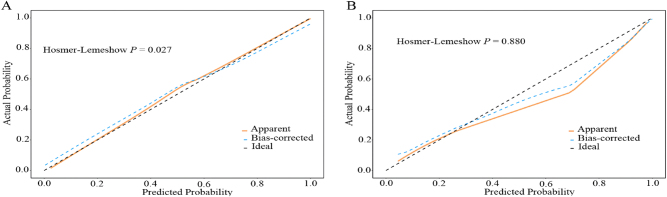
Calibration plots of the nomogram for discriminating obstetric antiphospholipid syndrome from healthy controls in the training set (A) and validation set (B). The *x*-axis represents the predicted probability of OAPS calculated by the nomogram; the *y*-axis represents the observed probability of OAPS. The solid diagonal reference line denotes a “perfect calibration” scenario, where the predicted probability of OAPS exactly matches the observed probability. The blue line represents the actual calibration performance of the nomogram: a closer fit between the blue line and the diagonal reference line indicates better agreement between predicted and observed probabilities. In the training set (A), the blue line is close to the diagonal, suggesting good calibration; in the validation set (B), the blue line shows a slight deviation but remains within a clinically acceptable range. A larger *P* value (>0.05) of Hosmer-Lemeshow test indicates no significant difference between predicted and observed probabilities, supporting good calibration; a smaller *P* value (<0.05) suggests potential calibration deviation.

**Figure 4:**
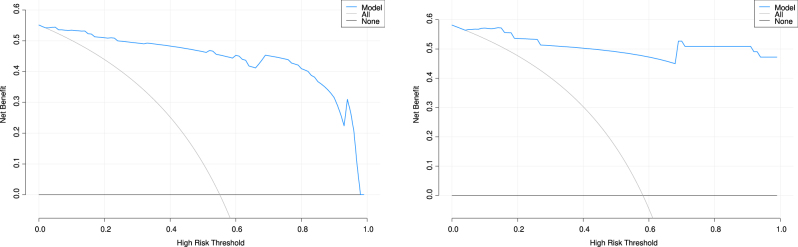
Decision curve analysis of nomogram for discriminating obstetric antiphospholipid syndrome from healthy controls in the training set (A) and validation set (B). The *x*-axis represents the threshold probability; the *y*-axis represents the net benefit. The blue line represents the nomogram; the black line represents the strategy of not intervening for any patient (None); the gray line represents the strategy of intervening for all patients (All). A model is clinically useful if its curve lies above the “None” and “All” lines.

A nomogram prediction model for OAPS, as shown in Figure 1, was constructed based on ANA, C3, aβ2-GPI, TAT, and Von Willebrand factor. Each variable corresponded to a point in the first line and the total points of all variables corresponded to the predicted value. For example, a woman with ANA of 1:100 (7 points), C3 of 1.0 (14 points), aβ2-GPI of 50 (18.5 points), TAT of 5 (25 points) and Von Willebrand factor of 100 (6.2 points) corresponded to a total point of 70.7. This value corresponded to a predicted value of 0.53. The ROC curve (Figure 2A, Table 6) showed that the area under the curve (AUC), sensitivity and specificity of the nomogram for predicting OAPS in the training set is 0.97 (95 % confidence interval [CI] 0.95–1.00), 0.89 (95 % CI 0.82–0.97), and 0.94 (95 % CI 0.89–1.00), respectively. The AUC, sensitivity and specificity of the nomogram model to predict OAPS in the validation set was 0.99 (95 % CI 0.98–1.00), 1.00 (95 % CI 1.00–1.00), and 0.88 (95 % CI 0.76–0.99), respectively (Figure 2B, Table 6). The Hosmer-Lemeshow test of the nomogram in the training set yielded a relatively small *P* value (*P* = 0.027), but the calibration plot visually showed good agreement between predicted and observed events. However, in the validation set, the Hosmer-Lemeshow test showed a larger *P* value (*P* = 0.880), but the visual fit of the calibration plot appeared suboptimal (Figure 3). The decision curve analysis (Figure 4) showed that the nomogram model provided more net benefits in both the training and validation set, supporting its potential clinical utility.

**Table 6:** Results of the receiver-operating characteristic curve for the nomogram to diagnose obstetric antiphospholipid syndrome in the training set and validation set.

	Cut-off	AUC (95 % CI)	Accuracy (95 % CI)	Sensitivity (95 % CI)	Specificity (95 % CI)	PPV (95 % CI)	NPV (95 % CI)
Training set	0.479	0.97 (0.95–1.00)	0.92 (0.86–0.96)	0.89 (0.82–0.97)	0.94 (0.89–1.00)	0.93 (0.86–1.00)	0.92 (0.85–0.98)
Validation set	0.479	0.99 (0.98–1.00)	0.93 (0.82–0.98)	1.00 (1.00–1.00)	0.88 (0.76–0.99)	0.85 (0.72–0.99)	1.00 (1.00–1.00)

AUC, area under the curve; CI, confidence interval; NPV, negative predictive value; PPV, positive predictive value.

### 3.5 Supplementary analysis of outliers in the validation set

**Figure 5:**
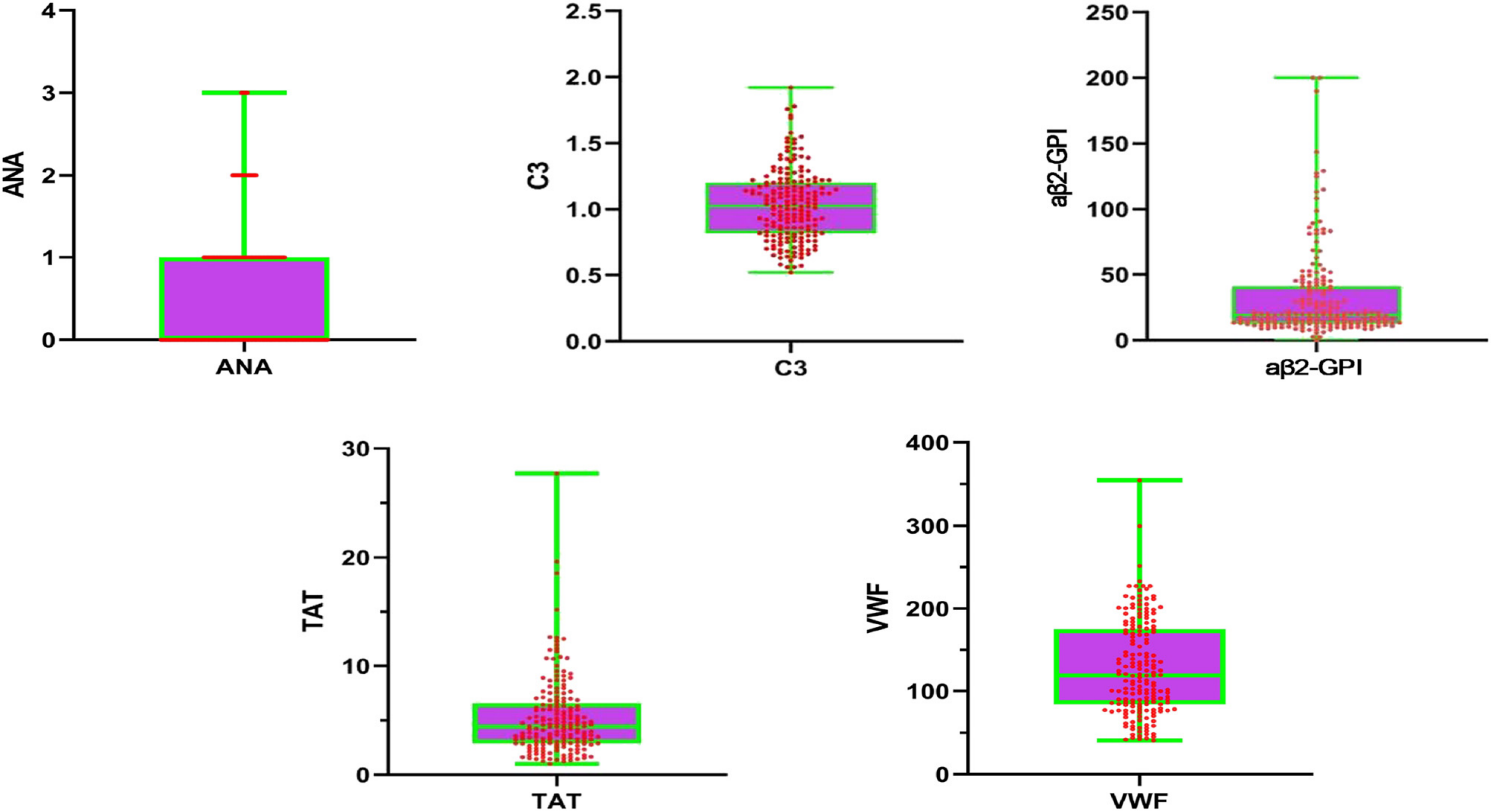
The value of ANA, C3, aβ2-GPI, TAT, and VWF in all patients. ANA, antinuclear antibody; aβ2-GPI, anti-β2 glycoprotein I antibody; TAT, thrombin-antithrombin complex; VWF, Von Willebrand factor.

In the external validation cohort of this study, outlier analysis was performed on core model variables including ANA, C3, aβ2-GPI, TAT, and Von Willebrand. In total, six outlier cases were identified, with several cases presenting extreme values in multiple parameters, most notably in aβ2-GPI and TAT (Figure 5 and Table 7). All these outliers were found among the OAPS group. As illustrated in Figure 5, these outliers distinguished themselves by exhibiting values far beyond the interquartile ranges of the validation set.

**Table 7:** The six outlier cases in the validation cohort.

Patient no.	ANA	C3	aβ2-GPI	TAT	VWF	Abnormal variables	Group
23	1:1,000	0.64	16.67	6.24	178.0	ANA	OAPS
59	1:100	1.00	127.21	11.94	354.3	aβ2-GPI, VWF	OAPS
64	1:100	1.20	129.23	27.70	97.0	aβ2-GPI, TAT	OAPS
69	1:320	0.83	200.00	3.25	98.0	aβ2-GPI	OAPS
75	1:100	0.67	143.68	4.78	131.5	aβ2-GPI	OAPS
97	1:100	0.56	45.10	18.55	81.2	TAT	OAPS

ANA, antinuclear antibody; aβ2-GPI, anti-β2 glycoprotein I antibody; OAPS, obstetric antiphospholipid syndrome; TAT, thrombin-antithrombin complex; VWF, Von Willebrand factor.

## 4 Discussion

In this single-center retrospective analysis of a mixed cohort of criteria-defined and non-criteria-defined OAPS, we identified predictors of OAPS and constructed a diagnostic nomogram. The results showed that levels of ANA, C3, aβ2-GPI, TAT, and Von Willebrand factor were significant predictors of OAPS, and the nomogram based on these variables had favorable diagnostic performance.

Earlier studies have revealed a positive association between APS, antiphospholipid antibodies and the risk of recurrent miscarriages [18], and the underlying mechanism may also be related to immune abnormalities and a pro-thromboembolic state. In a large population-based cohort study, Rhein et al. showed that the risk of late fetal loss, stillbirth, placental abruption, preeclampsia, fetal growth restriction, small for gestational age neonate, and low Apgar scores in OAPS increased by 14.3-, 4.9-, 3.8-, 3.4-, 3.3-, 3.0-, and 1.6-fold, respectively, as compared with the general obstetric population [19]. As demonstrated in this study, a number of coagulation indices, including APTT, D-dimer, TAT, and thromboelastography, exhibited significant differences between the OAPS and the healthy control group. This observation indicates that these indices may serve as promising diagnostic indicators. Logistic regression analysis identified ANA, C3, aβ2-GPI, and TAT as independent risk factors for OAPS, echoing previous notion that antiplatelet and anticoagulation therapy may help to reduce OAPS-related mortality [20].

Our study showed that the prevalence of positive ANA (1:100 or higher) was significantly higher in OAPS than in healthy controls, suggesting that ANA may serve as a convenient screening biomarker for OAPS. In addition, the work from Ottavi’s group also indicated that APS patients with ANA positivity would experience a higher rate of disease relapse, and thus the inclusion of ANA into the classification criteria would be helpful for tailoring individualized management [21].

Activation of the complement system by antiphospholipid antibodies has been shown to be an important etiopathogenesis in OAPS, which is supported by the findings of placental deposition of C3 and C4 [22], with correspondingly decreased plasma levels. The present study identified a decreased plasma C3 level as an independent risk factor for OAPS. Previous studies have established a mechanistically causal relationship between C3 activation and unfavourable pregnancy outcomes, including lower neonatal birth weight and premature delivery [23], 24].

The most recent update from the International Society on Thrombosis and Haemostasis recommends parallel testing of the dilute Russell’s viper venom time and APTT for the detection of lupus anticoagulant, based on the assumption that the Russell’s viper venom time is specific and less variable whereas the APTT test is more sensitive [25]. The silica clotting time is a type of phospholipid-dependent coagulation test by using silica as an activator and serves as an alternative to the APTT [26]. Our study identified no significant differences with regard to Russell’s viper venom time and silica clotting time between the OAPS and healthy control groups.

TAT is a surrogate biomarker for thrombin generation and has been identified as an early indicator of deep vein thrombosis [27]. Relevant studies have also reported elevated TAT levels in patients with systemic lupus erythematosus who have high IgM anticardiolipin antibody titers [28]. This finding is compatible with our observation that TAT is an independent risk factor for OAPS. As a relatively novel fibrolysis indicator, TAT level has also been demonstrated to be significantly elevated in pregnant women with early recurrent miscarriages [29], underscoring the utility of TAT in predicting pregnancy morbidities.

Von Willebrand factor is a plasma multi-domain glycoprotein that physiologically functions to promote homeostasis through platelet and subendothelial collagen adhesion [30]. An earlier study showed that the levels of active Von Willebrand factor are increased in APS and correlate with levels of aβ2-GPI IgG antibody [31]. IgG from APS patients has been demonstrated to stimulate the secretion of Von Willebrand factor, thereby potentially contributing to the thrombotic events observed in these patients [32]. Another *in vivo* study has shown that the inhibition of Von Willebrand factor degradation by a disintegrin and metalloproteinase with thrombospondin motifs 13 with aβ2-GPI antibodies may potentially elevate the risk of thrombosis [33]. Tian and colleagues identified elevated levels of Von Willebrand factor as a candidate biomarker for OAPS using 4D data-independent acquisition mass spectrometry [34], which is consistent with our result and supports its potential diagnostic utility in OAPS.

In this study, logistic regression analysis was successfully employed to identify the influencing factors, with statistically significant differences observed in ANA, C3, aβ2-GPI, TAT, and Von Willebrand factor. A nomogram model for diagnosing OAPS was constructed, and to ensure the accuracy of the prediction results, this study also employed the ROC curve, calibration curve, and decision curve analysis to evaluate the discriminative capability, accuracy, and net clinical benefit of the model. The results demonstrated that the nomogram exhibited favorable prediction accuracy and a high net clinical benefit, indicating that this prediction model demonstrated robust efficacy and substantial clinical utility. To the best of our knowledge, no specific nomogram for the diagnosis of OAPS has been reported, and only one study has established a nomogram that could predict the risk of ischemic stroke with an AUC of 0.793 in the validation cohort of patients with APS [35]. The nomogram designed in our study is a potential stratified screening tool for high-risk groups in obstetric and rheumatological settings to avoid overutilization and ensure efficiency. For some special population who might be misdiagnosed by current 2006 Sydney criteria or 2023 ACR/EULAR criteria for OAPS, the nomogram can be initiated at the first prenatal visit to guide further diagnostic and therapeutic decision. The nomogram’s input variables, including ANA titer, C3, aβ2-GPI, TAT, VWF are overlapped with existing routine prenatal or autoimmune workups, minimizing additional testing burden. To streamline data collection, we propose embedding a simplified data entry form into electronic health record systems. This form auto-populates laboratory values (e.g., C3, TAT) from the electronic health record’s laboratory module and allows clinicians to input ANA titer (e.g., 1:100, 1:320) and aβ2-GPI levels manually, reducing documentation time. Based on the total score calculated by the nomogram, patients can be stratified into different risk groups, with corresponding clinical actions. This stratification treatment ensures high-risk patients receive timely intervention while avoiding overtreatment of low-risk individuals. In summary, the nomogram is designed to be a pragmatic, guideline-aligned tool that enhances routine clinical practice. We believe the nomogram might be a valuable addition to clinical workflows, ultimately improving pregnancy outcomes for women at risk of OAPS. In the training set, the Hosmer-Lemeshow test yielded a small *P* value (*P* = 0.027), but the calibration plot visually showed good agreement between predicted and observed probabilities. However, in the validation set, the Hosmer-Lemeshow test showed a *P*-value of 0.880 (consistent with the expectation of good calibration), but the visual fit of the calibration plot appeared suboptimal. We speculated that these might be explained by three aspects: 1) Model complexity: The model might overfit the training data, resulting in unsatisfactory test results, but the calibration plot may look good; 2) Small sample size: In the case of a small sample, the test may lack sufficient statistical power to detect deviations; 3) Data distribution differences: The data distribution of the validation set may be different from that of the training set, leading to poor calibration effect. Therefore, the Hosmer-Lemeshow test may not be sufficient for evaluate the results. Further analysis of outliers in the validation set identified six outlier cases presenting extreme values in multiple parameters, most notably in aβ2-GPI and TAT. The presence of such cases may contribute to the calibration deviation observed for the nomogram model in the validation set, especially under the limitation of a small sample size, where individual extreme values can cause considerable fluctuation in predicted probabilities and model fit. Though introducing stricter inclusion or exclusion criteria for outliers might improve the statistical reproducibility and apparent stability of the model, it also risks omitting authentic yet highly heterogeneous cases that are relevant in real-world clinical scenarios, thereby compromising the generalizability of the model. In practical application, clinicians should combine the nomogram score with individual clinical characteristics for comprehensive judgment, rather than relying solely on model predictions. Establishing stricter inclusion criteria in future multi-center studies may balance the model’s stability and representativeness.

This study also suffers from several limitations that should be acknowledged. First, the study design is single-center cohort with a relatively small sample size and no external validation, thus extrapolation to patient populations with different characteristics should be cautious. A lack of population diversity in the training set may have reduced the nomogram’s inherent generalizability, as it could not capture variability in OAPS-related biomarkers or clinical phenotypes across different ethnicities, socioeconomic groups, or regional healthcare settings. To promote the application of the nomogram in diverse international populations, external validation across different ethnicities and regions would be critical to develop a broadly applicable diagnostic tool for OAPS. Another limitation is that other factors, such as previous pregnancy outcomes, were not considered, and relying exclusively on laboratory indicators alone may fail to fully capture the complexity and diversity of the pregnancy process.

## 5 Conclusions

In summary, this retrospective mixed cohort of criteria-defined and non-criteria-defined OAPS showed that ANA, C3, aβ2-GPI, TAT, and Von Willebrand factor are all crucial elements in the diagnosis of OAPS. The nomogram constructed based on these factors shows high predictive power and clinical applicability, thus facilitating the diagnosis and evaluation of patients with OAPS and the formulation of personalized treatment plans in a timely manner. However, further researches are still warranted to validate our conclusion in a larger sample size cohort conducted in multi-centers.

## References

[1] Alvarez D, Morales-Prieto DM, Cadavid AP (2023). Interaction between endothelial cell-derived extracellular vesicles and monocytes: a potential link between vascular thrombosis and pregnancy-related morbidity in antiphospholipid syndrome. Autoimmun Rev.

[2] Khormi AAM, Ba Gunaid M, Fayyad M, Mohrag M, AlAseeri AA (2024). Catastrophic antiphospholipid syndrome in a lupus patient with severe recurrent life-threatening clinical manifestations. J Med Cases.

[3] Banos N, Castellanos AS, Barilaro G, Figueras F, Lledo GM, Santana M (2022). Early prediction of adverse pregnancy outcome in women with systemic lupus erythematosus, antiphospholipid syndrome, or non-criteria obstetric antiphospholipid syndrome. J Clin Med.

[4] Wilson WA, Gharavi AE, Koike T, Lockshin MD, Branch DW, Piette JC (1999). International consensus statement on preliminary classification criteria for definite antiphospholipid syndrome: report of an international workshop. Arthritis Rheum.

[5] Miyakis S, Lockshin MD, Atsumi T, Branch DW, Brey RL, Cervera R (2006). International consensus statement on an update of the classification criteria for definite antiphospholipid syndrome (APS). J Thromb Haemostasis.

[6] Barbhaiya M, Zuily S, Naden R, Hendry A, Manneville F, Amigo MC (2023). ACR/EULAR antiphospholipid syndrome classification criteria. Ann Rheum Dis.

[7] Zhao Y, Huang C, Zhou Y, Qi W, Cai B, Hu C (2024). Performance validation of the 2023 American College of Rheumatology/European League against Rheumatism antiphospholipid syndrome classification criteria in an antiphospholipid syndrome cohort. J Thromb Haemostasis.

[8] Mısırcı S, Ekin A, Yağız B, Coşkun BN, Dalkılıç E, Pehlivan Y (2024). The validation of the 2023 ACR/EULAR antiphospholipid syndrome classification criteria in a cohort from Turkey. Diagnostics (Basel).

[9] Martínez-Taboada VM, Gómez AM, Del Barrio-Longarela S, Merino A, Comins-Boo A, López-Hoyos M (2024). Impact of the 2023 ACR/EULAR classification criteria in women with primary antiphospholipid syndrome during pregnancy. Diagnostics (Basel).

[10] Peng X, Tan X, Xing A (2023). An advanced understanding of the heterogeneous clinical features of “non-criteria” obstetric antiphospholipid syndrome: two case reports and a literature review. Front Immunol.

[11] Alijotas-Reig J, Esteve-Valverde E, Ferrer-Oliveras R, Saez-Comet L, Lefkou E, Mekinian A (2019). The European Registry on Obstetric Antiphospholipid Syndrome (EUROAPS): a survey of 1000 consecutive cases. Autoimmun Rev.

[12] Alijotas-Reig J, Esteve-Valverde E, Ferrer-Oliveras R, Saez-Comet L, Lefkou E, Mekinian A (2021). Corrigendum to: comparative study of obstetric antiphospholipid syndrome (OAPS) and non-criteria obstetric APS (NC-OAPS): report of 1640 cases from EUROAPS registry. Rheumatology (Oxford).

[13] Knight JS, Branch DW, Ortel TL (2023). Antiphospholipid syndrome: advances in diagnosis, pathogenesis, and management. BMJ.

[14] Yoon HJ (2019). Coagulation abnormalities and bleeding in pregnancy: an anesthesiologist’s perspective. Anesth Pain Med (Seoul).

[15] Rohilla M, Bhardwaj M, Jain V (2024). Perinatal outcomes in Indian women with Antiphospholipid Antibody Syndrome (APS): five year experience from a tertiary care centre. Eur J Obstet Gynecol Reprod Biol: X.

[16] Alijotas-Reig J, Esteve-Valverde E, Anunciación-Llunell A, Marques-Soares J, Pardos-Gea J, Miró-Mur F (2022). Pathogenesis, diagnosis and management of obstetric antiphospholipid syndrome: a comprehensive review. J Clin Med.

[17] Alijotas-Reig J, Esteve-Valverde E, Ferrer-Oliveras R, Saez-Comet L, Lefkou E, Mekinian A (2020). Comparative study of obstetric antiphospholipid syndrome (OAPS) and non-criteria obstetric APS (NC-OAPS): report of 1640 cases from the EUROAPS registry. Rheumatology (Oxford).

[18] Santos TDS, Ieque AL, de Carvalho HC, Sell AM, Lonardoni MVC, Demarchi IG (2017). Antiphospholipid syndrome and recurrent miscarriage: a systematic review and meta-analysis. J Reprod Immunol.

[19] Rhein AK, Rabinovich A, Abuhasira R, Lubaton-Barshishat S, Erez O (2024). Obstetric antiphospholipid syndrome carries an increased lifetime risk for obstetric and thrombotic complications – a population-based study. Res Pract Thromb Haemost.

[20] Depietri L, Veropalumbo MR, Leone MC, Ghirarduzzi A (2025). Antiphospholipid syndrome: state of the art of clinical management. Cardiovasc Drugs Ther.

[21] Ottavi M, Toulon P, Casolla B, Martis N (2024). Four clinical and biological phenotypes in antiphospholipid syndrome: a cluster analysis of 174 patients with antinuclear antibody tests. Front Immunol.

[22] Zhang Y, Jin S (2019). Mitigating placental injuries through up-regulating DAF in experimental APS mice: new mechanism of progesterone. Clin Exp Immunol.

[23] Alijotas-Reig J, Esteve-Valverde E, Llurba E, Gris JM (2019). Treatment of refractory poor aPL-related obstetric outcomes with TNF-alpha blockers: Maternal-fetal outcomes in a series of 18 cases. Semin Arthritis Rheum.

[24] Kim MY, Guerra MM, Kaplowitz E, Laskin CA, Petri M, Branch DW (2018). Complement activation predicts adverse pregnancy outcome in patients with systemic lupus erythematosus and/or antiphospholipid antibodies. Ann Rheum Dis.

[25] Devreese KMJ, de Groot PG, de Laat B, Erkan D, Favaloro EJ, Mackie I (2020). Guidance from the Scientific and Standardization Committee for lupus anticoagulant/antiphospholipid antibodies of the International Society on Thrombosis and Haemostasis: update of the guidelines for lupus anticoagulant detection and interpretation. J Thromb Haemostasis.

[26] Grypiotis P, Ruffatti A, Pengo V, Tonello M, Biasiolo A, Zamboni D (2006). Use of a new silica clotting time for diagnosing lupus anticoagulant in patients who meet the clinical criteria for antiphospholipid syndrome. J Clin Lab Anal.

[27] Lin Z, Sun H, Li D, Cai Z, Chen M, Zhang W (2022). Thrombin antithrombin complex concentration as an early predictor of deep vein thrombosis after total hip arthroplasty and total knee arthroplasty. BMC Musculoskelet Disord.

[28] Bugala K, Mazurek A, Gryga K, Komar M, Kopec G, Musial J (2018). Influence of autoimmunity and inflammation on endothelial function and thrombosis in systemic lupus erythematosus patients. Clin Rheumatol.

[29] Kjaergaard AB, Fuglsang J, Hvas AM (2021). Anti-Xa monitoring of low-molecular-weight heparin during pregnancy: a systematic review. Semin Thromb Hemost.

[30] Drakeford C, Aguila S, Roche F, Hokamp K, Fazavana J, Cervantes MP (2022). von Willebrand factor links primary hemostasis to innate immunity. Nat Commun.

[31] Huang S, Ninivaggi M, Chayoua W, de Laat B (2021). VWF, platelets and the antiphospholipid syndrome. Int J Mol Sci.

[32] Lindsey NJ, Dawson RA, Henderson FI, Greaves M, Hughes P (1993). Stimulation of von Willebrand factor antigen release by immunoglobulin from thrombosis prone patients with systemic lupus erythematosus and the anti-phospholipid syndrome. Br J Rheumatol.

[33] Ng CJ, McCrae KR, Ashworth K, Sosa LJ, Betapudi V, Manco-Johnson MJ (2018). Effects of anti-beta2GPI antibodies on VWF release from human umbilical vein endothelial cells and ADAMTS13 activity. Res Pract Thromb Haemost.

[34] Tian W, Shi D, Zhang Y, Wang H, Tang H, Han Z (2024). Deep proteomic analysis of obstetric antiphospholipid syndrome by DIA-MS of extracellular vesicle enriched fractions. Commun Biol.

[35] Song X, Fan Y, Jia Y, Li G, Liu M, Xu Y (2022). A novel aGAPSS-based nomogram for the prediction of ischemic stroke in patients with antiphospholipid syndrome. Front Immunol.

